# Factors in Pulmonary Embolus Diagnosis *via* CT Pulmonary Angiogram in Patients Undergoing Repair of Proximal Femur Fractures

**DOI:** 10.2174/1874325001812010236

**Published:** 2018-07-19

**Authors:** Peter Moriarty, Heather Moriarty, Michael Maher, James Harty

**Affiliations:** 1 Cork University Hospital, Wilton, Cork, Ireland; 2 Mater Misercordae University Hospital, Radiology Eccles St, Dublin, Ireland

**Keywords:** Hip fracture, Pulmonary emboli, CTPA, Pulmonary angiogram, Proximal, Femur

## Abstract

**Background:**

As imaging technology improves small Pulmonary Emboli (PE) of debatable clinical relevance are increasingly detected leading to higher numbers of patients receiving anticoagulation. Although PE are an important cause of morbidity and mortality in patients undergoing repair of proximal femur fractures, this cohort of patients are at increased falls risk and are therefore largely unsuitable for long term anticoagulant therapy.

**Objective:**

1. To review sequential Computed Tomography Pulmonary Angiograms (CTPA) performed in patients who underwent repair of proximal femur fractures at our institution. 2. To establish the perioperative CT imaging performed.

**Design:**

A retrospective cross sectional study of all patients undergoing proximal femur fracture repair at a single tertiary referral.

**Methods:**

The theatre database was interrogated to reveal all patients undergoing proximal femur fracture repair over a 28 month period from 01/01/12 to 07/04/14 inclusive. This was cross-referenced with the Picture Archiving Communication System (PACS) to establish all imaging undertaken in the perioperative period. CTPA studies performed within the time period of 1 week prior to and 6 months post proximal femur fixation were included. CTPA studies and reports were assessed for quality and findings. D-Dimer results, if performed within 72 hours of the CTPA study, were recorded.

**Results:**

1388 patients underwent neck of femur fracture repair in the 28-month study period. Of this cohort 71 CTPA studies were performed in 71 patients (5.2%) with a mean age of 77.8 years (range 38 - 100). 53 (74.6%) of studies were negative for embolus and 17 (23.9%) studies revealed clot in a pulmonary artery (1 saddle embolus, 2 main pulmonary artery emboli, 7 lobar vessel emboli, 2 segmental artery emboli, 5 subsegmental emboli). Overall PE detection rate was 1.2% of our total study population. In all 71 studies, Houndsfield Unit (HU) in the main pulmonary artery (PA) was >200; which is considered to be of satisfactory quality to assess for segmental pulmonary emboli. 32% of patients had D Dimer levels performed, however no relationship with presence of PE on CTPA was demonstrated.

**Conclusion:**

The rate of positive CTPA studies in patients undergoing proximal femur fracture repair is 23.9% in our patient population, comparing favorably to published data. This is likely to reflect good compliance with prevention measures at ward level. D-Dimer results are unreliable for PE prediction.

## 1 INTRODUCTION

Orthopaedic patients with neck of femur fractures can have significant morbidity and mortality; indeed the Department of Health published the national 30 day mortality rate following hip fracture surgery was 2.46 deaths per 100 cases, ranging from 1.43 deaths per 100 cases to 3.68 deaths per 100 case. However, when adjusted for age no statistically significant differences were observed at the 95% level of confidence. Importantly, the national age-standardised in-hospital mortality following hip fracture surgery has reduced over the 10 years period from 2001 to 2010 [1].

PE is a serious form of Venous Thromboembolism (VTE), and leads to the hospitalisation or death of over 300,000 people in Europe each year. It is not uncommon diagnosis in this patient group [2, 3]. Timely and appropriate diagnosis and management impact on morbidity and mortality.

In a study by Cushman *et al*. the 28-day fatality rate was 11% in patients with VTE [1]. Other factors affecting prognosis for thromboembolic disease include age, co-morbidities and race in certain studies [4].

The International Cooperative Pulmonary Embolism Registry was to establish mortality rates of PE and to identify factors associated with mortality. The 3-month overall mortality rate was 17.4%; 45.1% of these deaths were considered due to PE. Systolic arterial hypotension, congestive heart failure, cancer, tachypnoea, right-ventricular hypokenisis on echocardiography, chronic obstructive pulmonary disease, and age >70 years were associated with increased mortality risk in patients with PE [5].

The risk of PE appears to be elevated in the extended post operative period, not just the immediate post operative period. Epidemiologic data from the mid 1990s demonstrated that cumulative 90-day symptomatic venous thromboembolism rates after hip fracture surgery was 1.9% [6], reported in other studies of patients undergoing orthopedic surgery similarly at 2.7%-2.8% [7, 8].

Techniques employed with the intention to reduce the risk of PE include anti-coagulation or anti-platelet agents, early mobilization, amongst other methods. According to Khan *et al*, the use of LMWH for prophylaxis of 10-14 days, is expected to prevent 13 venous thromboembolism per 1,000 patients undergoing major orthopedic surgery, if a baseline risk of 1% for PE and 1.8% for symptomatic DVT is assumed [9].

## 2 DIAGNOSIS OF PULMONARY EMBOLUS

Acute PE is recognized as a challenging clinical diagnosis to make and is potentially fatal if undiagnosed. Given the potentially high incidence of falls particularly in a trauma patient population, and thus the potential for devastating haemorrhage if anti-coagulated, the accurate and timely diagnosis is of utmost importance and can allow discussion on how to best manage these patients in whom the decision as to whether to anti-coagulate or not can often be challenging [10]. There are many factors which may influence outcome in patients with PE, particularly with ever improving diagnostic accuracy, and increasing imaging resolution, which may now identify emboli which would not have previously been diagnosed. For patients in the acute setting with suspected PE, CTPA is the test of choice for most clinicians. In a meta-analysis by Safriel *at al*. the overall sensitivity and specificity for CTPA was 74.1% and 89.5% with a range of 57-100% and 68-100%, respectively, for the detection of PE [11]. It it is estimated that 1-2% of all patients who attend the emergency department undergo CTPA for the investigation of PE [12]. The increased demand for CTPA may be driven by the perception that it produces a definite result: positive or negative. Other advantages of CTPA include its high sensitivity and accuracy, and also the detection of alternative pathologies.

The accuracy of CTPA has been well established with a meta-analysis suggesting that with the aid of a pretest probability score, CTPA is unlikely to result in false negative diagnosis [13]. The interpretation of a filling defect leads to a diagnosis of PE, which in most cases commits the patient to anticoagulation. The inter-observer agreement in the radiological interpretation of CTPA for PE is approximately 87.2%-93% [14, 15]. A 2005 study by Chartr *et al*. examined the intra observer agreement between radiologists of different subspecialties [16]. This study interestingly evaluated for discordance based on location lobar, intralobar and segmental arteries showed a trend for decreasing concordance from centrally to more peripherally located disease, with an agreement of 83% for lobar pulmonary embolism and 57% for segmental pulmonary embolism. This trend was also identified in the PIOPED study where agreement was 98% for lobar pulmonary embolism, 90% for segmental pulmonary embolism and 66% for subsegmental pulmonary embolism [17]. These results have been reflected in other studies [18, 19]. There can be marked variance in the distribution of burden of embolus within the lungs, and the clinical syndrome this may produce. Obstruction of a main pulmonary artery may cause significant morbidity, whereas a single vessel obstruction in a subsegmental artery may not be of clinical importance. Interestingly the latter studies were blinded radiologists to the clinical information. It has yet to be determined what impact clinical information, or lack thereof, in the post traumatic population, has on inter observer agreement. Quality of the CTPA study is an important determining factor for interpreter confidence and the reporter’s ability to give an accurate and clinically useful report. For those examinations concluded to be indeterminate, poor contrast opacification contributes to 40% of examinations and motion artefact to 74%. It is suggested that the optimal opacification in the main pulmonary artery should be 200-250 Houndsfield units [20, 21].

## 3 AIM

1. We aim to establish the number of neck of femur fracture patients who had CTPAs over a 28 months period from a single major trauma center.
2. To establish the rates of positive and negative studies and the rate of equivocal studies.
3. To quantify the location/burden of emboli in the positive studies and examine the main factors influencing image interpretation in the equivocal studies.
4. To assess the type, frequency and indication for perioperative CT imaging (excluding CTPA) performed in this patient cohort undergoing major orthopedic surgery.

## 4 METHODS

The intranet Cork University Hospital (CUH) theatre database was interrogated using search terms “hip”, “neck of femur” and “IM nail”. This revealed 1388 patients undergoing NOF fracture repair over a 28 month period from 01/01/12 to 07/04/14 inclusive. The patient medical record numbers were then entered into the hospitals PACS system to establish the imaging undertaken in the perioperative period.

CTPA studies performed within the time period of 1 week prior to surgery and 6 months post neck of femur repair were included. CTPA studies and reports were scrutinized for subjective and objective study quality including Houndsfield units >200, result and incidental findings.

In our patient group we assessed studies for the location of PE (pulmonary trunk, main pulmonary artery, segmental pulmonary artery, subsegmental pulmonary artery) and also, and in particular in subsegmental disease, to establish the number of vessels involved.

The medical record numbers of the patients who underwent CTPA studies were subsequently entered into the hospital information system, and D-dimer results, if performed within 72 hours of the CTPA study, were recorded. The absolute value of D-Dimer was assessed for correlation with the level of PE, if present on CTPA.

The PACS database was interrogated for concurrent imaging in the perioperative period. CT brain studies performed within the 7 days preceding, or the 30 days post op were recorded for indication and acute findings. CT studies of the pelvis (including orders for CT ‘hip’ and CT ‘femur’ were noted including the indication and main imaging findings.

Data were recorded by a single observer in an annonomised spreadsheet using Microsoft Excel 2013 (v15.0). The recorded data was analyzed using IBM SPSS Statistics version 2015

## 5 RESULTS

### 5.1 Patient Demographics

**Fig. (1):**
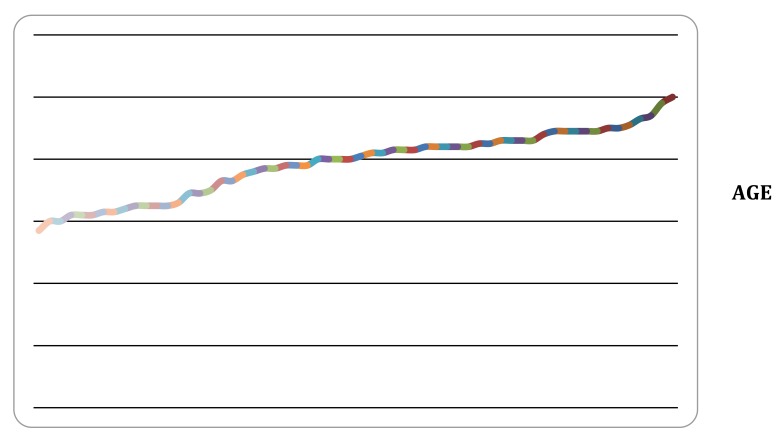
Line chart depicting the increasing rates of older patients undergoing CTPA’s for investigation of pulmonary embolus.

**Fig. (2):**
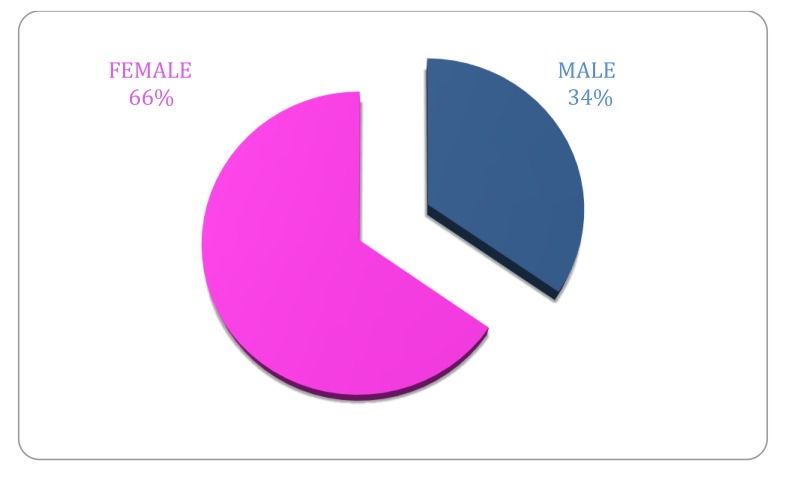
Pie chart representing the the breakdown of females and males undergoing CTPA’s during the study period. The majority of patients were females (n=47, 66%) compared to males (n=24, 34%).

Within the study period 71 patients underwent CTPA for evaluation of pulmonary embolus. 47 were female and 24 were male (Fig. **2**). There was no data available for 5 patients. The mean age of patients undergoing CTPA was 77.8 years of age (range 38-100 years, Fig. **1**).

### 5.2 Number of Patients

#### 5.2.1 Type of Fracture and Surgical Intervention

**Fig. (3):**
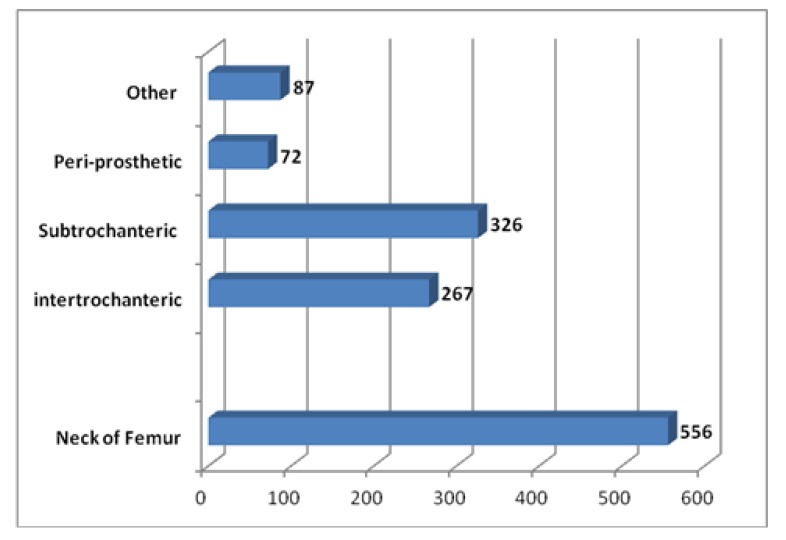
Horizontal bar chart highlighting the type of fractures diagnosed in patients presenting acutely during the study period. The majority of patients had neck of femur fractures followed by subtrochanteric and intertrochanteric fractures.

The majority of patients in this cohort presented acutely with Neck of femur fractures (Fig. **3**). This accounted for 40% of the study group. A similar number of patients were diagnosed with extracapsular fractures (n=593). Furthermore, 72 patients presented peri-prosthetic fractures requiring intervention and 87 patients were labeled as other which included fractures secondary to metastatic deposits or impending fractures secondary to metastatic lesions. Moreover a subset of these patients included peri-prosthetic dislocations.

**Fig. (4):**
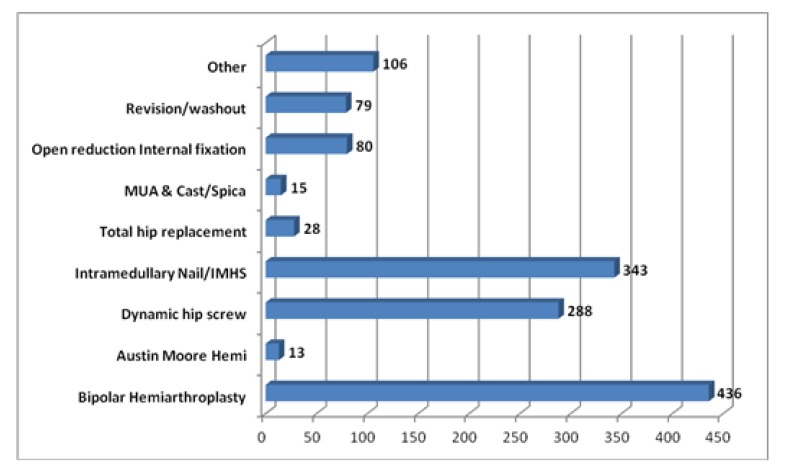
Horizontal bar chart displaying type of surgical fixation carried out during the study period. A large proportion of patients underwent bipolar hemiarthroplasty followed by intramedullary nail/IMHS and dynamic hip screw.

Different surgical approaches and fixation techniques were undertaken throughout the study period (Fig. **4**). The type of fixation undertaken was dependant on the fracture type. A large number of patients underwent a bipolar hemiarthroplasty (n=436) which is in keeping with the fact the majority of the patient (n=556) present with neck of femur fractures. The second most common fixation device undertaken was an intermedullary nail followed by a dynamic hip screw (n= 343, 288, respectively). These approaches are typically carried out in patients with extracapsular hip fractures with are classified as subtrochanteric (n=326) and intertrochanteric (267) in this study.

### 5.3 Anesthesia

**Fig. (5):**
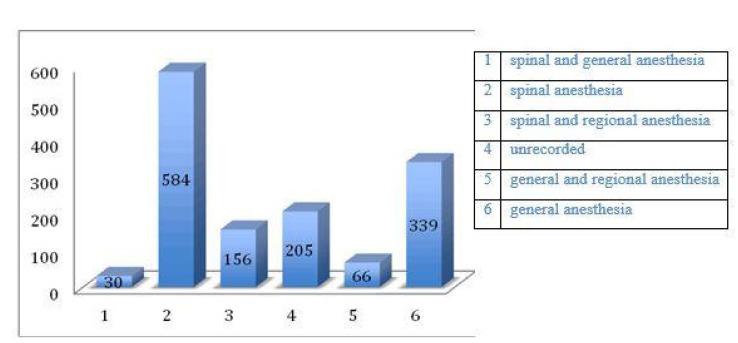
Bar chart highlighting the type of anaesthesia used on patients undergoing proximal neck of femur fracture during the study period.

Of the 1388 patients undergoing neck of femur fracture repair 30 patients had spinal and general anaesthesia (2%, Fig. **7**). 584 had spinal anesthesia alone (42%). 156 patients had spinal and regional anesthesia (11%). In 205 patients (14%) the type of anaesthesia was unrecorded. 66 patients (4.7%) had general and regional anaesthesia. 339 patients (24%) had general anesthesia alone (Fig. **5**).

Of the patients who underwent CTPA studies 3 patients (4%) had spinal and regional anesthesia, 26 patients (36%) had spinal and general anaesthesia, 40 patients (56%) had spinal anesthesia alone.

### 5.4. CTPA Results

**Fig. (6):**
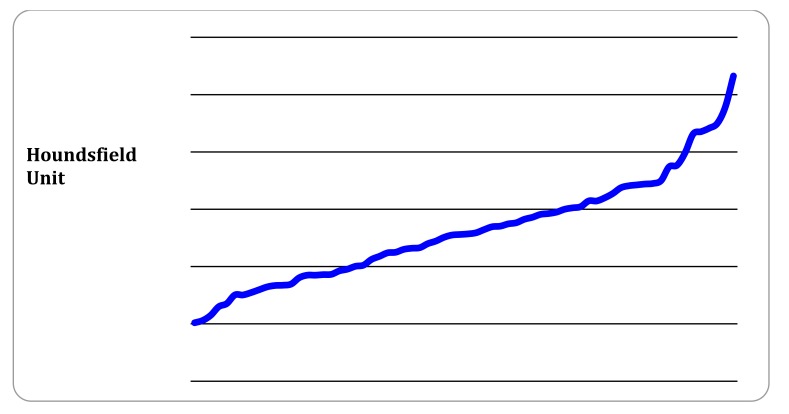
Pie chart demonstrating CTPA results of patients being investigated for pulmonary embolus.

CTPA studies at this institution were performed with a bolus tracking trigger, with a region of interest manually drawn over the main pulmonary artery by the CT radiographer. Of the 1388 patients included in our analysis, 71 patients had CTPA studies (5.2%). These studies were analyzed for the number of positive, negative and equivocal studies (Fig. **6**). 53 studies were negative for embolus (74.6% of studies). 17 studies were positive for a thrombus in a pulmonary artery (23.9%). One study was equivocal for subsegmental PE. This gives an overall detected pulmonary embolus rate of 1.2% in our neck of femur fracture surgical population.

## 6 STUDY QUALITY

**Fig. (7):**
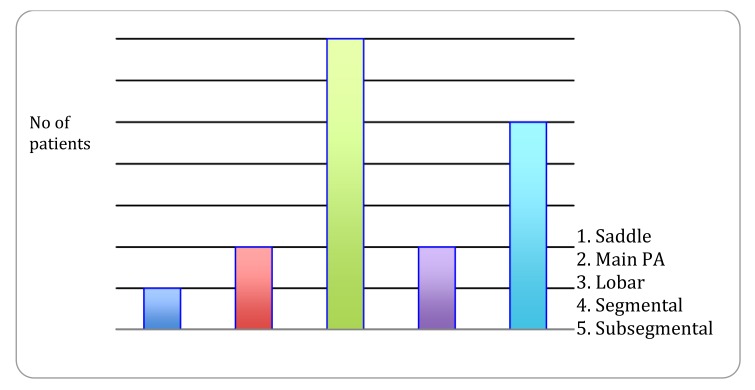
Line chart demonstrating Houndsfield units of all CTPA’s performed during the study period. All CTPAs had greater than 200 units ensuring that they were all of effective diagnostic quality.

In all 71 studies, Houndsfield Unit (HU) in the main PA was greater than 200 units (Fig. **7**). This is deemed acceptable in the literature, and generally taken as good opacification of the main PA.

In 16 cases, the CTPA reports described the study as slightly limited or suboptimal 12 studies documented that respiratory motion artifact was present however the majority of these were still of satisfactory quality to assess for sub segmental PE.

While all studies were deemed sufficient quality to evaluate for thrombus in the pulmonary trunk and in the main, lobar and segmental vessels, one study was reported as equivocal, with two equivocal sub segmental vessels specifically identified, however this was also reported as negative for segmental or higher order PE. In a small number of CTPA studies where the evaluation of sub segmental pulmonary arteries was limited it was most commonly due to respiratory motion artifact. Furthermore, in one patient the study was slightly limited due to marked dilation of the pulmonary trunk and central PA’s secondary to pulmonary arterial hypertension causing contrast pooling centrally. A CTPA was deemed indeterminate in the context of large burden of metastatic thoracic disease and large bilateral pleural effusions.

## 7 BURDEN OF DISEASE

**Fig. (9):**
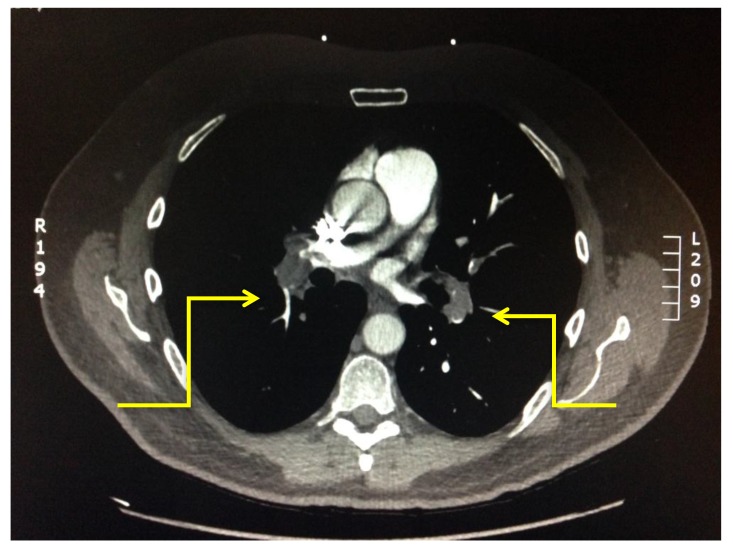
Axial CTPA view displaying a segmental pulmonary embolising the right upper lobe highlighted by yellow arrow.

**Fig. (10):**
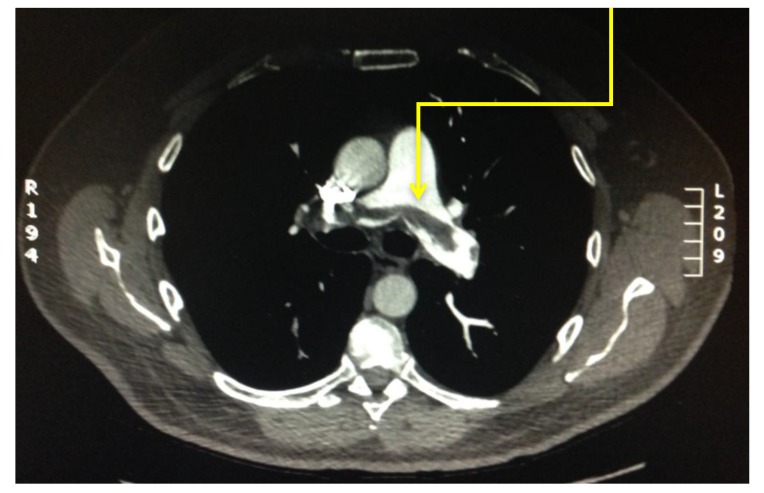
Axial view of a patients CT demonstrating a right main and left lobar pulmonary emboli outlined by two yellow arrows.

**Fig. (11):**
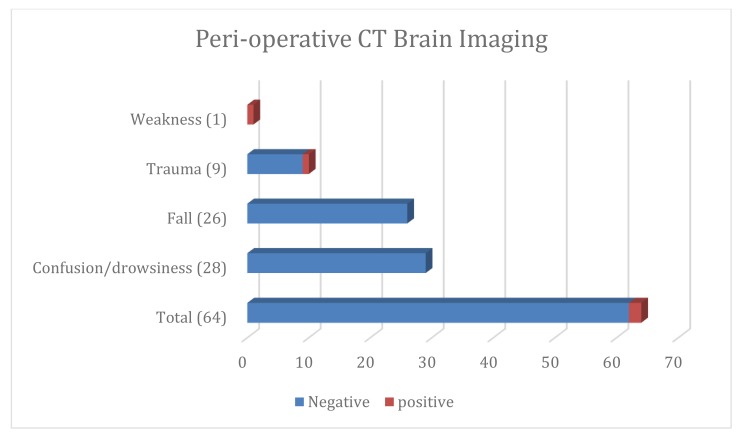
Axial view of patient’s CT with a saddle pulmonary embolus demonstrated by the yellow arrow.

The level of vessel affected by PE is represented in Fig. (**8**). Of the 17 studies which were found to be positive for pulmonary emboli, one was a saddle pulmonary embolus (Fig. **11**) obstructing the bilateral main pulmonary arteries. There were two patients with thrombus in the main pulmonary arteries (Fig. **10**), one unilateral and the other bilateral. Seven patients had embolus identified in their lobar arteries, and two with clot in segmental arteries (Fig. **9**). There were 5 patients with subsegmental pulmonary emboli identified. Of these five patients one had multiple vessels in both lungs, involving 5 segments. A further two patients had thrombus identified within only 2 subsegmental vessels. Two patients had thrombus identified within a single subsegmental vessel, one of which had imaging characteristics of a chronic embolus, the other appeared acute in nature.

## 8 D DIMER

D dimer is a much mooted test, and has proved a sensitive test to exclude the presence of clot. Although all patients were post op, and thus were likely to have raised D Dimer [4, 3].

32% of patients had D Dimers performed in the 72 hours preceding or following their CTPA. While only 1 was found to be within normal range < 0.5% at this institution the subsequent scan yielded a negative result.

## 9 CONCURRENT IMAGING

### 9.1 CT Brain

**Fig. (12):**
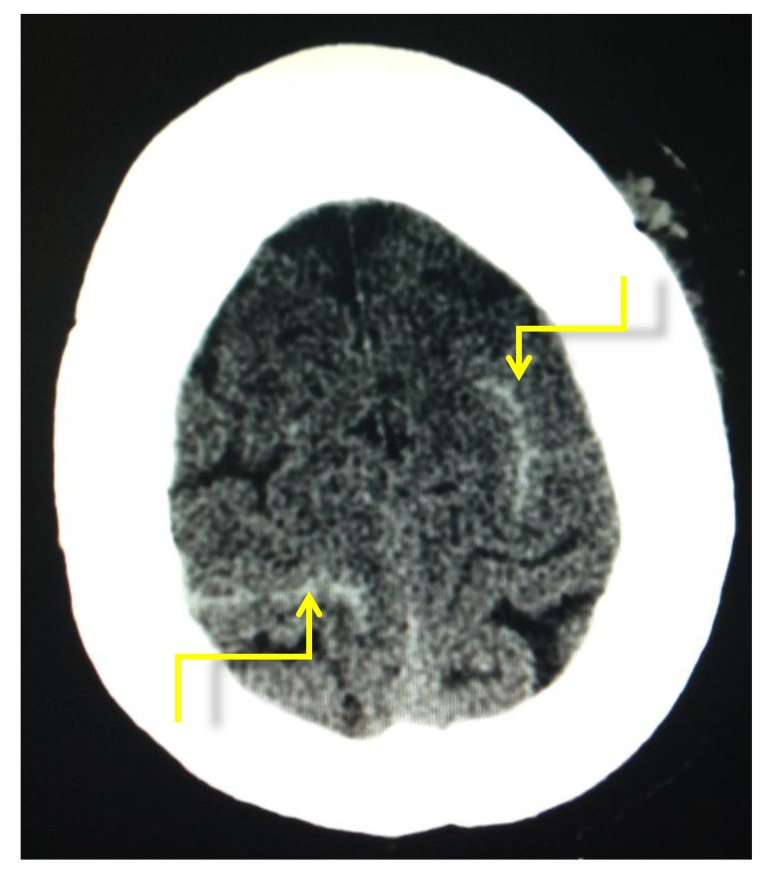
Bar chart displaying indications for patients undergoing concurrent CT brain. The majority of patients underwent CT brain for work-up of confusion/drowsiness or fall in the peri-operative period.

**Fig. (13):**
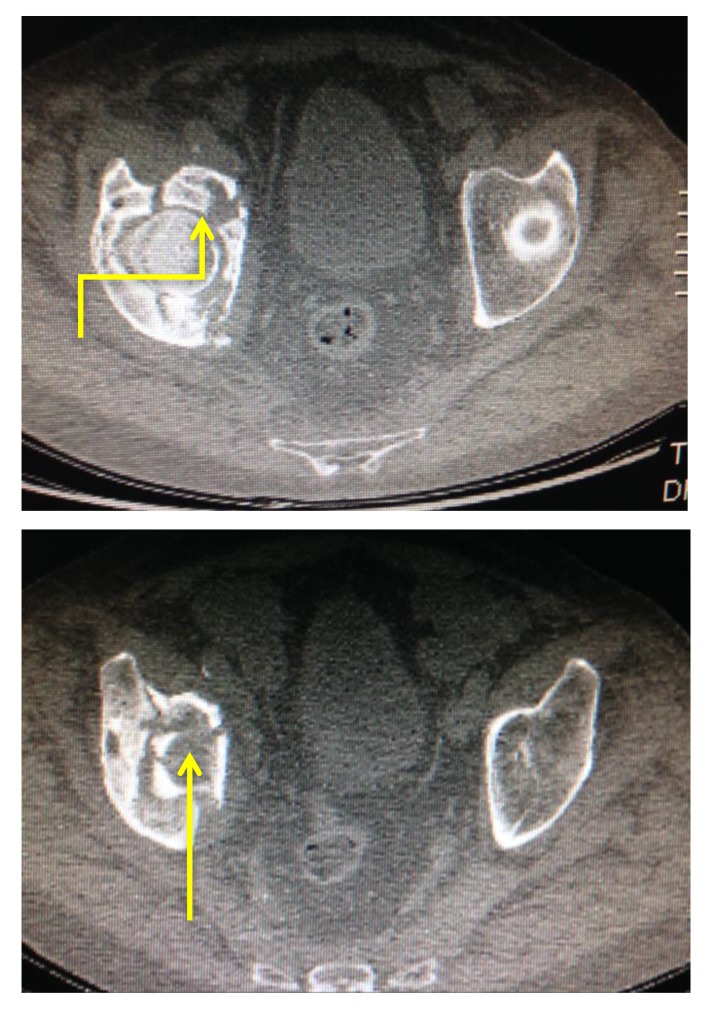
Sulcal subarachnoid haemorrhage overlying the bilateral frontal lobes at the vertex post major trauma (road traffic accident) in a patient who also sustained a neck of femur fracture.

**Fig. (14):**
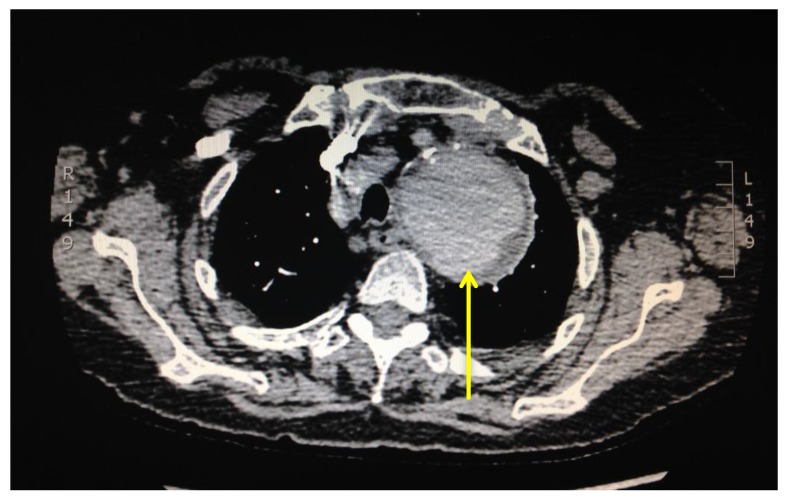
CT pelvis performed for pre operative planning in a comminuted right acetabular/femoral head fracture highlighted by the yellow arrow.

Of the 599 patients from May 2013 to June 2014, 64 had perioperative CT brains. The clinical indication (Fig. **12, 13**) included 29 were performed for confusion, 26 for falls, nine as part of a trauma work-up and one for left sided weakness. Overall positivity rate was 3.25% (n=2). Of the 54 scans that were performed for confusion/falls no acute pathology was revealed. One CT brain study performed for left sided weakness revealed subacute infarction and a second performed pre operatively revealed subarachnoid and intraparenchymal haemorrhage (Fig. **14**) post road traffic accident in which the patient also sustained a neck of femur fracture.

### 9.2 CT Pelvis

**Fig. (15):**
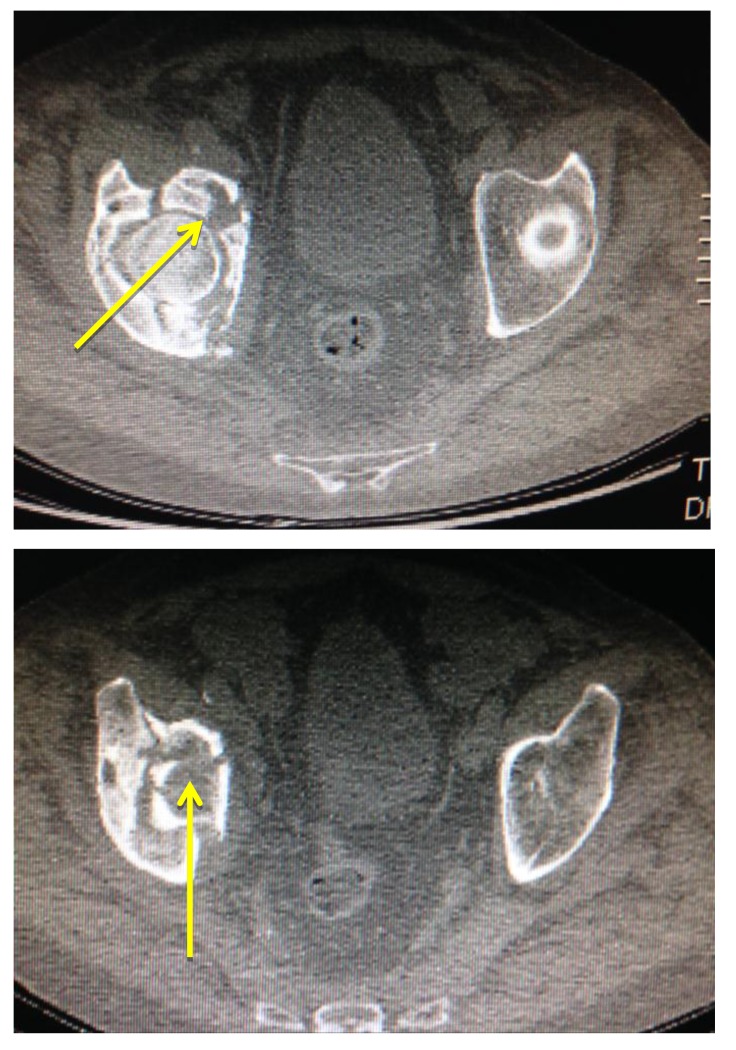
Incidental findings of patients under CTPAs in this study - A 7 cm incidental thoracic aortic aneurysm as outlined by yellow arrow.

2.5% percent of patients in this study had pre-operative pelvic CT. Seven of these were to establish if there was a fracture present as plain radiography was inconclusive. This was for various reasons such as a true occult fracture or patient movement during acquisition, or in one case a lytic lesion was identified on radiographic imaging and was subsequently proven to be a metastatic deposit from a lung cancer primary. The indication in four cases was for pre-operative planning, often concerning complex fractures (Fig. **15**). A single patient had an intermediate study post initial fixation with a view to further surgery.

## 10 INCIDENTAL FINDINGS

In 41 patients, incidental findings were noted on CTPAs. The most common finding was consolidation in 15 patients and pleural effusions in 14 patients. Unexpected significant findings were reported in 6 patients, including 2 patients with rib fractures, and one patient with a 7cm aortic arch aneurysm (Fig. **15**). Further evaluation with triphasic CT imaging was recommended in 1 patient, in whom a dilated pancreatic duct was identified. Subsequent to the patient undergoing a triphasic CT pancreas an ultimate diagnosis of side branch IPMN was agreed. Interval follow up imaging of incidental findings was recommended in 4 patients, all of whom had pulmonary nodules which required follow up.

## 11 DISCUSSION

The incidence of PE following orthopaedic trauma has been reported at rates as high as 27.8% throughout the literature [22]. In this current study of 1388 patients undergoing repair of proximal femur fractures, 71 patients (5.2%) required a CTPA for investigation of an underlying pulmonary embolus. Of the 71 patients, it was observed that one third of patients were male and two thirds female which is in keeping with published data in the Irish Hip Fracture Database Preliminary Report 2013 [1]. Of 71 CTPAs performed, 17 studies were positive for a thrombus in a pulmonary artery (23.9%) representing an overall detected pulmonary embolus rate of 1.2% in our neck of femur fracture surgical population. Given the major clinical consequences a pulmonary embolism may have, particularly in this vulnerable, largely elderly patient group, many of whom will have significant co morbidities, the diagnosis of a PE, where there is clinical concern is of utmost import. It has been suggested in the literature that thromboembolic events can explain a large proportion of morbidity and mortality association with fixation after an acute hip fracture in elderly patients. Of the 10% reported mortality rates in this group of patients approximately 0.5% are attributed to pulmonary embolus [23]. The incidence of positive studies in this current study compares favorably to published data *at a1.* 2%.

Clinical probability tools are used on a ward level to determine the probability of a patient having a PE prior to undergoing CTPA. Such scoring systems include the Wells score or the modified Geneva scoring system and have been widely investigated to assess their predictive value in determining PE risk in different subgroups of hospitalized patients [24-27]. The majority of studies have analysed these scoring tools in non traumatic patients and have proven to be of some benefit. Despite these finding data suggests that these scoring tools are not significantly predictive of PE in trauma patients admitted to the Orthopaedic trauma service [28, 29]. Within this study serum D-Dimer levels were analysed in a subset of patients undergoing CTPA. The presence of an elevated D Dimer was correlated with a positive finding of a PE on CTPA, however it is not possible to draw conclusions from this due to the confounding factors of trauma and surgery. There was no correlation with level of elevation of D Dimer and the presence of a PE observed in this study. It is also suggested that the time to analyze serum D-dimer may, in this patient group, contribute an unnecessary delay.

Location of embolus within the pulmonary arterial system was analyzed in all 17 positive cases within our study cohort. One patient had a saddle pulmonary embolus occluding the bilateral main pulmonary arteries, demonstrating a high degree of disease burden. Moreover, there were two patients with thrombus in the main pulmonary arteries, one unilateral and the other bilateral. Seven patients had embolus identified in their lobar arteries, and two with clot in segmental arteries. There were 5 patients with subsegmental pulmonary emboli identified. The bolus tracking technology in our patient group has allowed excellent opacification of the main pulmonary artery in all cases. Where there is peripheral vascular hypo-attenuation, there is often evidence of intrinsic patient factors - such as pulmonary artery dilatation or right heart failure, and ensuing with central puddling of the intra-arterial contrast bolus, which may not be possible to correct pre testing. Outcomes in PE can vary dramatically and disease burden is a major contributor to mortality and morbidity. Miller *et al*. derived an index for the quantification of clot burden for conventional angiography [30]. A study by Wu *et al*. in 2003 demonstrated a strong correlation between clot burden and patient mortality [31]. Their data suggested that ‘PE index’ which encompasses clot burden is a significant predictor of patient outcome (*P* = .002). Furthermore the authors displayed that by using a cutoff of 60%, the PE index was used to identify 52 of 53 (98%) patients who survived and five of six (83%) patients who died. They conclude from the evidence presented that quantification of clot burden with CTPA rather than a false/positive result is an important predictor of patient death in the setting of PE. A further study by Van der Meer *et al*. demonstrated that >40% obstruction of the pulmonary vasculature correlated with an 11.2-fold risk of death due to PE [32]. Which such positive findings surrounding embolus burden many authors attempted to adapt the Miller Index using different anatomical models of the pulmonary vasculature.

Within this current study, 76 patients (5.6%) had further imaging within 30 days of their proximal neck of femur fixation. The majority were CT brains performed for confusion, falls or investigation of cerebrovascular accident (CVA). 3.25% of CT brains were positive for intracerebral haemorrhage. This is similarly reported throughout the literature due cohort of the patients involved and also the possibility of underlying dual pathology with proximal neck of femur fracture. Studies have been performed to investigate the incidence of CVA in patients with hip fractures and also predictive scoring systems to identify high risk patients. A study by Popa *et al*. performed a population based cohort study of patients undergoing operative hip fixation. In their study 1606 patients underwent operative intervention over a 14 years’ period with an 3.9% incidence of CVA [33]. This reflects similar findings in our current study. Interestingly, the authors highlight that previous documented stroke and acute hip fractures were the strongest positive predictors of stoke in a multivariate analysis. It is documented that the incidence of stroke is increased in patients undergoing hip fracture repair (1.5% at 30 days and 5.5% at 1 year) compared to patients undergoing elective hip replacement surgery (0.6% at 30 days and 1.5% at 1 year). This likely reflects the differences in patient cohorts and the acute nature of hip fracture surgery, whereas patients undergoing elective surgery are likely to be optimized pre-operatively. Published data from Lawrence *et al*. estimate that early event rates of CVA or Transient Ischemic Attack (TIA) is approximately 1% following hip fracture [34]. This study also analysed patients within the first 30 days of admission however included a younger population group.

It is well understood that with newer radiological modalities and improved imaging techniques that patients undergoing investigations such as CTPAs are being increasingly diagnosed with incidental findings. Within our cohort, 41 patients were noted to have incidental finding on CTPAs. The most common finding was consolidation followed by pleural effusions. Unexpected significant findings were reported in 6 patients. A study by Rashid *et al*. reported that in 191 patients undergoing CTPA’s that 150 patients had alternative of incidental findings. Despite having a 21% positive rate of underlying PE, a large number of patients were diagnosed with lower respiratory tract infection, heart failure and lung malignancy. This was a similar finding in our study. A study by Thompson *et al* reported a rate of incidental findings of 33.4% in emergency CT scans performed [35]. Their study examined all modalities of imaging and found that CT abdomens had the highest incidental findings. They report incidental findings in 46.2% of CT thorax performed. Furthermore, they report that, similar to our study, patients age greater than 60 years were more likely to have incidental findings.

### 12 LIMITATIONS

The study is limited by patient numbers, despite the high number of neck of femur fracture operations; the number of patients undergoing CTPA studies is small. It is also the reporting radiologist’s prerogative to comment on study quality and to which level pulmonary emboli are confidently excluded, however on assessment of the 70 CTPA studies in conjunction with the radiologist report it is considered likely that these factors were commented on, if significant, in all cases.

## 13 FUTURE WORK AND POTENTIAL CLINICAL APPLICATIONS

The use of clinical decision supports in the general population has been shown to reduce monthly CTPA use by 12.5%. This makes use of an algorithm based on the Modified Geneva Score, PERC score and D-Dimers. Use of previously validated scores may not be effective in the orthopaedic population as many of the criteria are fulfilled by nature of the patient’s injury. We consider the possibility of a checklist for Orthopaedic patients risk stratified based on CTPA positivity rates per fracture type. Furthermore, a 100% negative rate of perioperative CT brain scans for the indication of Falls/Confusion may suggest significant waste of resources and ionizing radiation which, in our opinion, warrants further investigation/discussion. Although the number of negative D Dimer results were low (1 case), this subsequently yielded a negative CTPA. This should be taken into consideration before employing more expensive, resource heavy investigations.

## CONCLUSION

The rate of positive CTPA studies in patients undergoing proximal femur fracture repair is 23.9% in our patient population, comparing favorably to published data. This is likely to reflect good compliance with prevention measures at ward level. D-Dimer results are unreliable for PE prediction.

## Figures and Tables

**Fig. (8):**
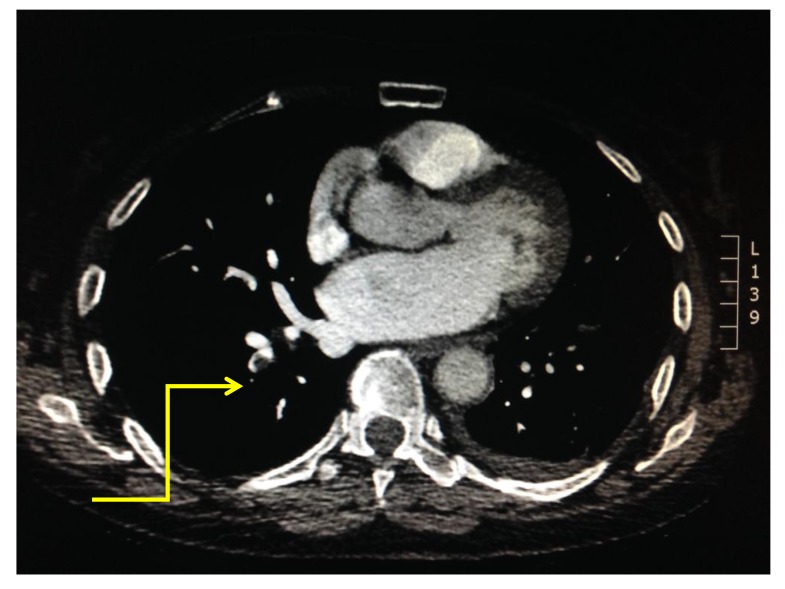
Bar chart highlighting the level of vessel affected by embolic disease amongst patients with a positive CTPA during the study period. Distribution of embolic event within the pulmonary vasculature.

## References

[1] http://health.gov.ie/wp-content/uploads/2014/03/Health-Care-Quality-Indicators-in-the-Irish-Health-System.pdfHealth Care Quality Indicators in the Irish Health System Examining the Potential of Hospital Discharge Data using the Hospital Inpatient Enquiry System. 2017.

[2] Cohen A.T., Agnelli G., Anderson F.A., Arcelus J.I., Bergqvist D., Brecht J.G., Greer I.A., Heit J.A., Hutchinson J.L., Kakkar A.K., Mottier D., Oger E., Samama M.M., Spannagl M., VTE Impact Assessment Group in Europe (VITAE) (2007). Venous thromboembolism (VTE) in Europe. The number of VTE events and associated morbidity and mortality.. Thromb. Haemost..

[3] Cushman M., Tsai A.W., White R.H., Heckbert S.R., Rosamond W.D., Enright P., Folsom A.R. (2004). Deep vein thrombosis and pulmonary embolism in two cohorts: The longitudinal investigation of thromboembolism etiology.. Am. J. Med..

[4] Siddique R.M., Siddique M.I., Connors A.F., Rimm A.A. (1996). Thirty-day case-fatality rates for pulmonary embolism in the elderly.. Arch. Intern. Med..

[5] Goldhaber S.Z., Visani L., De Rosa M. (1999). Acute pulmonary embolism: Clinical outcomes in the international cooperative pulmonary embolism registry (ICOPER).. Lancet.

[6] White R.H., Zhou H., Romano P.S. (2003). Incidence of symptomatic venous thromboembolism after different elective or urgent surgical procedures.. Thromb. Haemost..

[7] White R.H., Romano P.S., Zhou H., Rodrigo J., Bargar W. (1998). Incidence and time course of thromboembolic outcomes following total hip or knee arthroplasty.. Arch. Intern. Med..

[8] Bjørnarå B.T., Gudmundsen T.E., Dahl O.E. (2006). Frequency and timing of clinical venous thromboembolism after major joint surgery.. J. Bone Joint Surg. Br..

[9] White R.H., Romano P.S., Zhou H., Rodrigo J., Bargar W. (1998). Incidence and time course of thromboembolic outcomes following total hip or knee arthroplasty.. Arch. Intern. Med..

[10] Kahn S.R., Houweling A.H., Granton J., Rudski L., Dennie C., Hirsch A. (2014). Long-term outcomes after pulmonary embolism: current knowledge and future research.. Blood Coagul. Fibrinolysis.

[11] Safriel Y., Zinn H. (2002). CT pulmonary angiography in the detection of pulmonary emboli: A meta-analysis of sensitivities and specificities.. Clin. Imaging.

[12] Kline J.A., Courtney D.M., Kabrhel C., Moore C.L., Smithline H.A., Plewa M.C., Richman P.B., O’Neil B.J., Nordenholz K. (2008). Prospective multicenter evaluation of the pulmonary embolism rule-out criteria.. J. Thromb. Haemost..

[13] Quiroz R., Kucher N., Zou K.H., Kipfmueller F., Costello P., Goldhaber S.Z., Schoepf U.J. (2005). Clinical validity of a negative computed tomography scan in patients with suspected pulmonary embolism: A systematic review.. JAMA.

[14] Courtney D, Miller C, Smithline H

[15] Shaham D., Heffez R., Bogot N.R., Libson E., Brezis M. (2006). CT pulmonary angiography for the detection of pulmonary embolism: Interobserver agreement between on-call radiology residents and specialists (CTPA interobserver agreement).. Clin. Imaging.

[16] Chartr C (2005).

[17] PIOPED Investigators (1990). Value of the ventilation/perfusion scan in acute pulmonary embolism. Results of the prospective investigation of pulmonary embolism diagnosis (PIOPED).. JAMA.

[18] Stein P.D., Fowler S.E., Goodman L.R., Gottschalk A., Hales C.A., Hull R.D., Leeper K.V., Popovich J., Quinn D.A., Sos T.A., Sostman H.D., Tapson V.F., Wakefield T.W., Weg J.G., Woodard P.K., PIOPED II Investigators (2006). Multidetector computed tomography for acute pulmonary embolism.. N. Engl. J. Med..

[19] Courtney D, Miller C, Smithline H

[20] Stein P.D., Athanasoulis C., Alavi A., Greenspan R.H., Hales C.A., Saltzman H.A., Vreim C.E., Terrin M.L., Weg J.G. (1992). Complications and validity of pulmonary angiography in acute pulmonary embolism.. Circulation.

[21] Stein P.D., Fowler S.E., Goodman L.R., Gottschalk A., Hales C.A., Hull R.D., Leeper K.V., Popovich J., Quinn D.A., Sos T.A., Sostman H.D., Tapson V.F., Wakefield T.W., Weg J.G., Woodard P.K., PIOPED II Investigators (2006). Multidetector computed tomography for acute pulmonary embolism.. N. Engl. J. Med..

[22] Tornetta P., Bogdan Y. (2012). Pulmonary embolism in orthopaedic patients: Diagnosis and management.. J. Am. Acad. Orthop. Surg..

[23] Pulmonary Embolism Prevention Trial Collaborative Group (2000). Prevention of fatal post operative pulmonary embolism and deep vein thrombosis with low dose aspirin; Pulmonary Embolism Prevention (PEP) tial.. Lancet.

[24] Le Gal G., Righini M., Roy P.M., Sanchez O., Aujesky D., Bounameaux H., Perrier A. (2006). Prediction of pulmonary embolism in the emergency department: The revised Geneva score.. Ann. Intern. Med..

[25] Wells P.S., Ginsberg J.S., Anderson D.R., Kearon C., Gent M., Turpie A.G., Bormanis J., Weitz J., Chamberlain M., Bowie D., Barnes D., Hirsh J. (1998). Use of a clinical model for safe management of patients with suspected pulmonary embolism.. Ann. Intern. Med..

[26] Wells P.S., Anderson D.R., Rodger M., Ginsberg J.S., Kearon C., Gent M., Turpie A.G., Bormanis J., Weitz J., Chamberlain M., Bowie D., Barnes D., Hirsh J. (2000). Derivation of a simple clinical model to categorize patients probability of pulmonary embolism: Increasing the models utility with the SimpliRED D-dimer.. Thromb. Haemost..

[27] Geersing G.J., Erkens P.M., Lucassen W.A., Büller H.R., Cate H.T., Hoes A.W., Moons K.G., Prins M.H., Oudega R., van Weert H.C., Stoffers H.E. (2012). Safe exclusion of pulmonary embolism using the Wells rule and qualitative D-dimer testing in primary care: prospective cohort study.. BMJ.

[28] Wolf S.J., McCubbin T.R., Feldhaus K.M., Faragher J.P., Adcock D.M. (2004). Prospective validation of Wells Criteria in the evaluation of patients with suspected pulmonary embolism.. Ann. Emerg. Med..

[29] Runyon M.S., Webb W.B., Jones A.E., Kline J.A. (2005). Comparison of the unstructured clinician estimate of pretest probability for pulmonary embolism to the Canadian score and the Charlotte rule: A prospective observational study.. Acad. Emerg. Med..

[30] Miller G.A., Sutton G.C., Kerr I.H., Gibson R.V., Honey M. (1971). Comparison of streptokinase and heparin in treatment of isolated acute massive pulmonary embolism.. BMJ.

[31] Wu A.S., Pezzullo J.A., Cronan J.J., Hou D.D., Mayo-Smith W.W. (2004). CT pulmonary angiography: Quantification of pulmonary embolus as a predictor of patient outcome—initial experience..

[32] van der Meer R.W., Pattynama P.M., van Strijen M.J., van den Berg-Huijsmans A.A., Hartmann I.J., Putter H., de Roos A., Huisman M.V. (2005). Right ventricular dysfunction and pulmonary obstruction index at helical CT: Prediction of clinical outcome during 3-month follow-up in patients with acute pulmonary embolism.. Radiology.

[33] Popa AS, Rabinstein AA, Huddleston PM, Larson DR, Gullerud RE, Huddleston JM (2009). Predictors of Ischemic Stroke After Hip Operation: A Population-Based Study.. Journal of hospital medicine : An official publication of the Society of Hospital Medicine.

[34] Lawrence V.A., Hilsenbeck S.G., Noveck H., Poses R.M., Carson J.L. (2002). Medical complications and outcomes after hip fracture repair.. Arch. Intern. Med..

[35] Rashid M, Murthy M, Pocock A, Varia R. (2013). Incidence of positive yield and alternative diagnoses made after imaging performed for suspected acute pulmonary embolism.. Pulmonary embolic. disease..

